# Protection against *Babesia* infection by red blood cell PIEZO1 activation

**DOI:** 10.1128/spectrum.04114-25

**Published:** 2026-07-29

**Authors:** Kai Wengelnik, Rakhee Lohia, Anais Paveyranne, Jordy Le Guet, Gregor P. Jose, Stéphane Azoulay, Eric Honoré, Dominique Douguet

**Affiliations:** 1 LPHI, University of Montpellier, CNRS, Inserm, Montpellier, France; 2 University Côte d'Azur, ICN, CNRS, Nice, France; 3 University Côte d’Azur, IPMC, CNRS, Inserm U1323, UMR7275, Labex ICST, Valbonne, France.; Uniwersytet Medyczny w Bialymstoku, Bialystok, Poland; Lanzhou Veterinary Research Institute, Lanzhou, China

**Keywords:** erythrocyte, mechanosensitive ion channel, Piezo1, *Babesia divergens*, babesiosis

## Abstract

*Babesia divergens* are tick-borne apicomplexan parasites infecting red blood cells of domestic and wild animals in Europe, as well as humans with important health burden. Previous findings indicate that PIEZO1 gain-of-function inherited mutations confer a significant protection against cerebral malaria. In this study, we further demonstrate that several Yoda1 analogs promoting PIEZO1 opening prevent *B. divergens* infection of human red blood cells *in vitro*. We identify several compounds with improved specificity for *B. divergens,* as compared to *Plasmodium falciparum*.

**IMPORTANCE:**

*Babesia* are apicomplexan parasites transmitted by tick bites that mainly infect animals, but some species such as *Babesia divergens* also infect people with dramatic outcomes. Babesiosis represents a threat for bovine production and is also an emerging human infectious disease in endemic regions. Like the malaria parasite *Plasmodium*, *Babesia* infects red blood cells (RBC) and causes hemolysis. Protection is currently based on drug treatments, tick control strategies, and animal vaccination. However, the indiscriminate use of prophylactic agents directly targeting the parasite is at risk to cause drug resistance, as observed for malaria. Here, we show that PIEZO1 activation in human RBC, taking advantage of newly synthesized Yoda1 analogs with enhanced efficacy and lower cytotoxicity, confers *in vitro* protection against *Babesia* infection.

## INTRODUCTION

Babesiosis generally progresses asymptomatically or induces mild flu-like symptoms. However, in asplenic or immunocompromised patients, the disease can become fulminant and fatality rates can be as high as 20% (1). *B. microti* in the USA and *B. divergens* in Europe are the most common species responsible for over a thousand of severe cases per year (2). A possible therapeutic option is to target the host instead of the parasite to reduce the risk of pharmacological resistance of the parasite (3). Eventually, both strategies targeting at the same time the parasite and the host could be used in combination. In red blood cells (RBC), opening of the mechanosensitive cationic channel PIEZO1 (4) causes a calcium influx leading to RBC dehydration (5). Inherited gain-of-function (GOF) mutations (6, 7) and pharmacological sensitization *in vitro* of PIEZO1 by Yoda1 (8) have been shown to confer significant protection against malaria infection of human RBC (9). Here, we synthetized new compounds that prevent *B. divergens* infection of human RBC with lower toxicity and higher specificity.

## RESULTS

### Antibabesial activity of previously reported PIEZO1 chemical sensitizers

**Fig 1:**
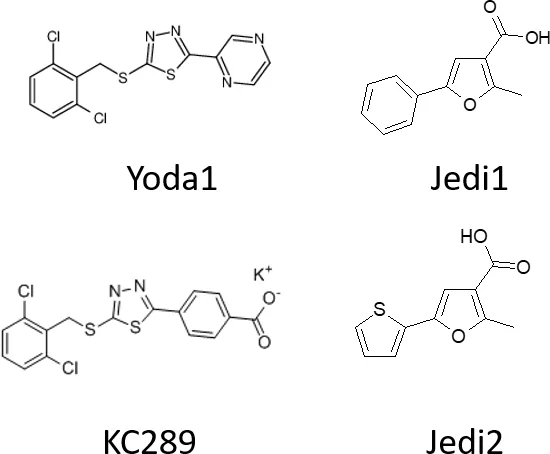
Structure of previously reported PIEZO1 chemical sensitizers.

Growth inhibition of *B. divergens* was performed on *in vitro* parasite cultures in human RBC. In parallel, the potential cytotoxicity of these compounds was investigated against the mammalian lymphoblast erythroid cell line K562 that grows as a cell suspension similar to *P. falciparum* culture in human RBC. A selectivity index (SI, ratio of toxicity over activity) above 10 reflects favorable properties. Yoda1 was active against *B. divergens* (IC_50_ of 1.7 µM) with a SI of 24.7 (Table 1 and Fig. 1). In contrast, only high concentrations of Jedi1, Jedi2 (10), or KC289 (11) showed antibabesial activity similar to what was observed with *P. falciparum* (9).

**TABLE 1:** Antibabesial activity of previously published chemical sensitizers of PIEZO1

Compd^*a*^	*B. divergens* IC_50_ [μM]^*b*^	Cytotoxicity IC_50_ [μM]^*c*^ SI
Yoda1	1.7 ± 0.6 (*n* = 12)	42 ± 30 (*n* = 12) SI = 24.7
Jedi1	475 ± 106 (*n* = 2)	>2,000 SI > 4.2
Jedi2	525 ± 176 (*n* = 2)	>2,000 SI > 3.8
KC289	16.3 ± 2.5 (*n* = 3)	113.3 ± 23.1 (*n* = 3) SI = 7

^*a*^ Compound name.

^*b*^ IC_50_ value of antibabesial activity (mean ± SD and the number of independent experiments).

^*c*^ IC_50_ value of the cytotoxicity against the mammalian cell line K562. The selectivity index (SI) is the ratio of IC_50_ values of cytotoxicity over the activity against *B. divergens*.

### Antibabesial activity of novel Yoda1 analogs

**Fig 2:**
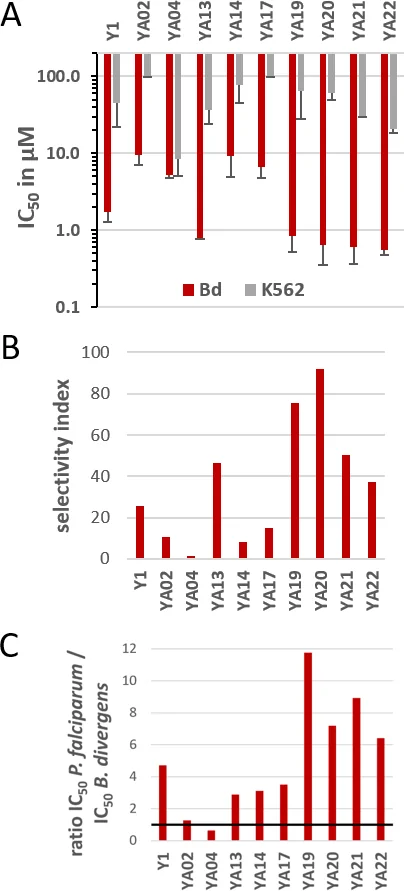
Activities of commercial analogs (YA02–YA17) and newly synthesized molecules (YA19–YA22) with lower toxicity and higher specificity. (**A**) IC_50_ values in µM against *B. divergens* (Bd) and toxicity to K562 mammalian cells (mean ± SEM, *n* = 2 to 15). (**B**) Selectivity index (SI, ratio of cytotoxicity over activity, *n* = 2 to 15). (**C**) Ratio of IC*_50_* for *P. falciparum* over *B. divergens* (*n* = 2 to 15), performed in parallel experiments. Y1, Yoda1.

**Fig 3:**
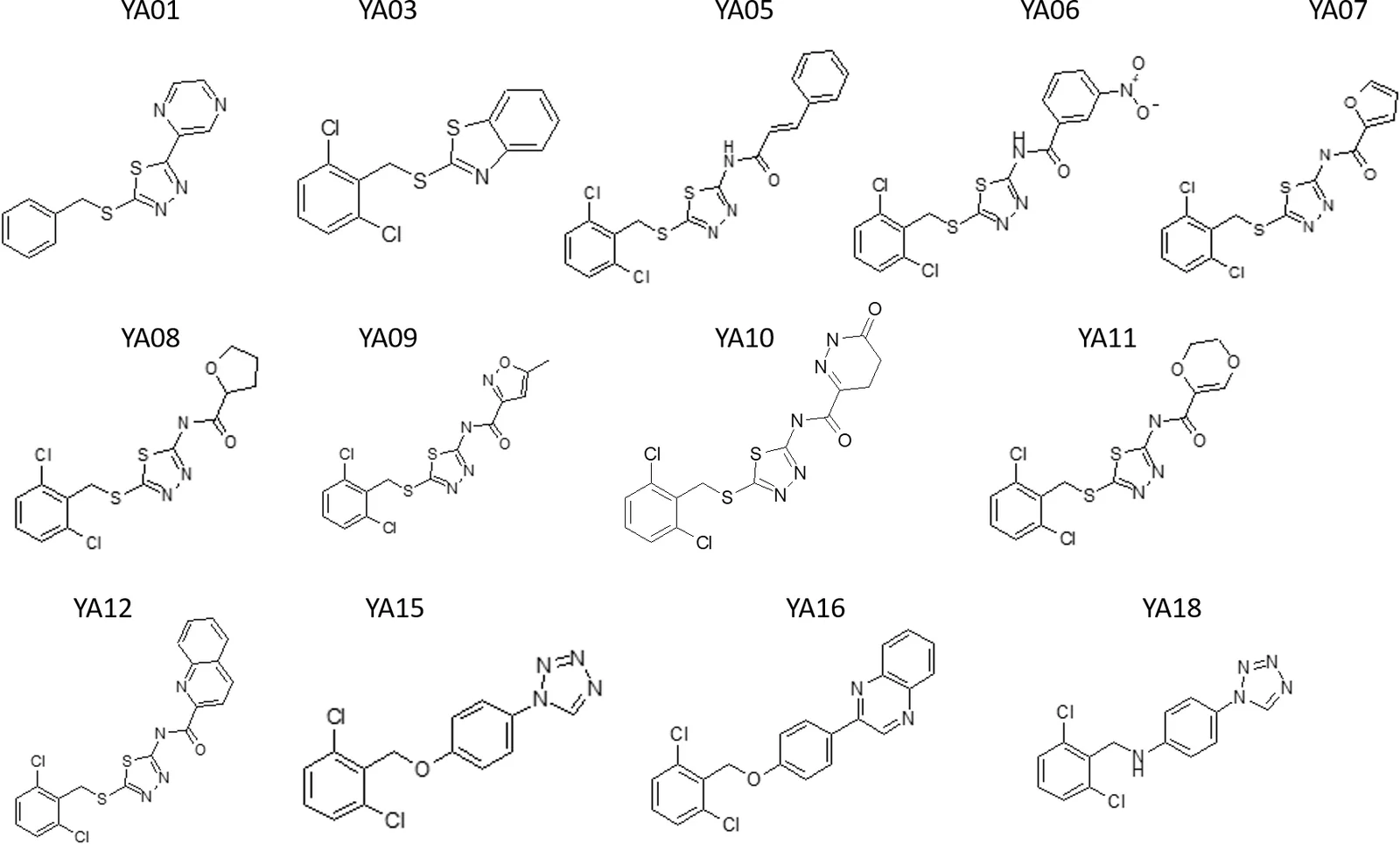
Structure of commercial analogs with low potency.

**Fig 4:**
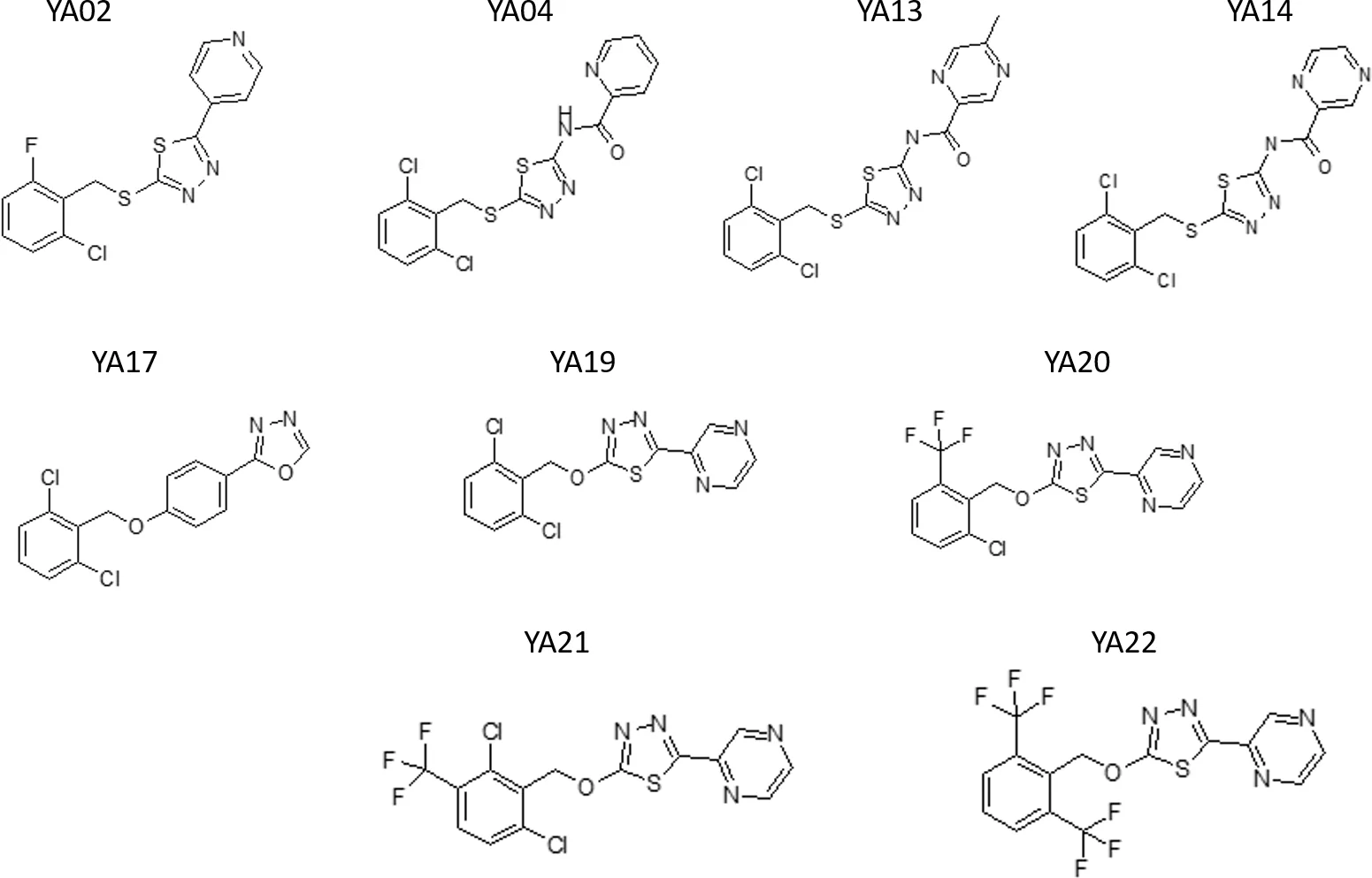
Chemical structures of the compounds (YA02–YA17) and newly synthesized molecules (YA19–YA22).

**Fig 5:**
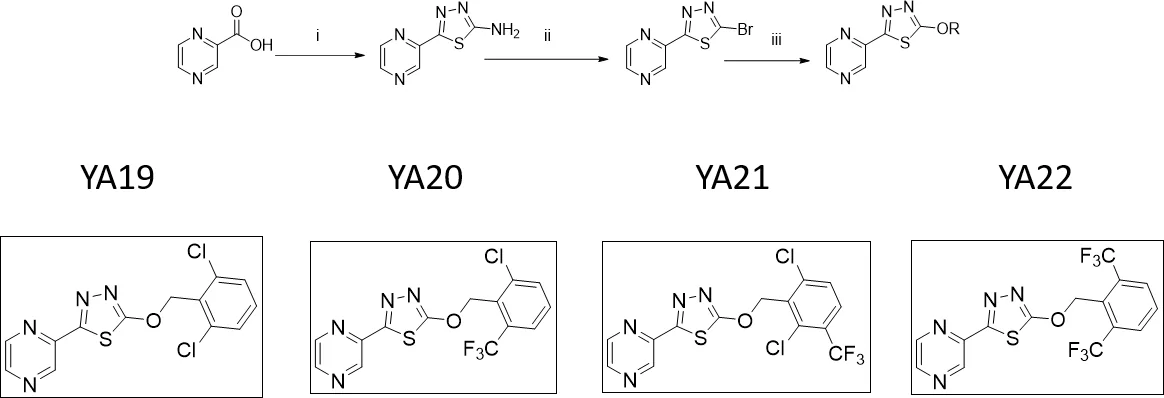
Chemical synthesis of Yoda1 analogs: (i) A/ (COCl)₂/catalytic DMF/CH_2_Cl_2_, 4 h, RT, then hydrazinecarbothioamide, B/ H_2_SO_4_, 3 h, 100°C; (ii) CuBr_2_/t-BuNO_2_/MeCN, 65°C, o.n.; (iii) NaH/KI/CuO/ROH.

Next, we evaluated commercial analogs of Yoda1 (termed YA). Replacements of the dichlorophenyl (YA01 and YA02), pyrazine (YA02 and YA04 to YA14), thiadiazole (YA03 and YA15 to YA17), and thioether (YA15 to YA18) were tested (Table 2, Fig. 2 to 4). Five of the 18 analyzed compounds showed antibabesial activity with an IC_50_ of less than 10 µM (YA02, YA04, YA13, YA14, and YA17, Fig. 2). The activity of the ether-based compound YA17 prompted us to synthesize Yoda1-like molecules with this linker (Fig. 5). Chemical syntheses led to the production of YA19 (Fig. 4) displaying a low IC_50_ of 0.85 μM and an enhanced SI of 75.3 (Fig. 2). While the two o-chlorines are critical for PIEZO1 activation (12), replacement by trifluoromethyl groups in YA20 and YA22 led to submicromolar inhibitors with elevated SI of 92 and 37.5, respectively (Fig. 2). Finally, the addition of a trifluoromethyl group at the m-position in YA21 (Fig. 4) showed an improved IC_50_ of 0.61 μM and enhanced SI of 50 (Fig. 2). In summary, we synthesized four molecules, YA19 to YA22, with submicromolar IC_50_ against *B. divergens* and elevated SI values (Fig. 2A and B). Notably, YA19–YA22 also showed superior protection of human RBC against *B. divergens* infection, as compared to *P. falciparum* (Fig. 2C).

**TABLE 2:** Antibabesial activity of commercial analogs with low potency

Compd^*a*^	B. divergens IC_50_ [µM]^*b*^	Cytotoxicity IC_50_ [µM]^*c*^ SI
YA01	>100 (*n* = 1)	>100
YA03	>100 (*n* = 1)	>100
YA05	>100 (*n* = 1)	>50
YA06	60 (*n* = 1)	>100 SI = 1.7
YA07	>100 (*n* = 1)	>100
YA08	60 (*n* = 1)	>100 SI > 1.7
YA09	60 (*n* = 1)	>100 SI > 1.7
YA10	17.2 ± 13 (*n* = 3)	>100 SI > 5.8
YA11	17.4 ± 4.6 (*n* = 4)	65 ± 7 (*n* = 2) SI = 3.7
YA12	26.7 ± 9.9 (*n* = 3)	50 (*n* = 2) SI = 1.9
YA15	24 ± 1 (*n* = 2)	36 ± 5 (*n* = 2) SI = 1.5
YA16	17.3 ± 16.2 (*n* = 3)	>50 (*n* = 2) SI > 2.9
YA18	55 (*n* = 1)	>100 SI > 1.8

^*a*^ Compound name.

^*b*^ C_50_ value of antibabesial activity (mean ± SD and the number *n* of independent experiments).

^*c*^ IC_50_ value of the cytotoxicity against the mammalian cell line K562. SI, selectivity index.

### Activation of PIEZO1 by novel Yoda1 analogs

**Fig 6:**
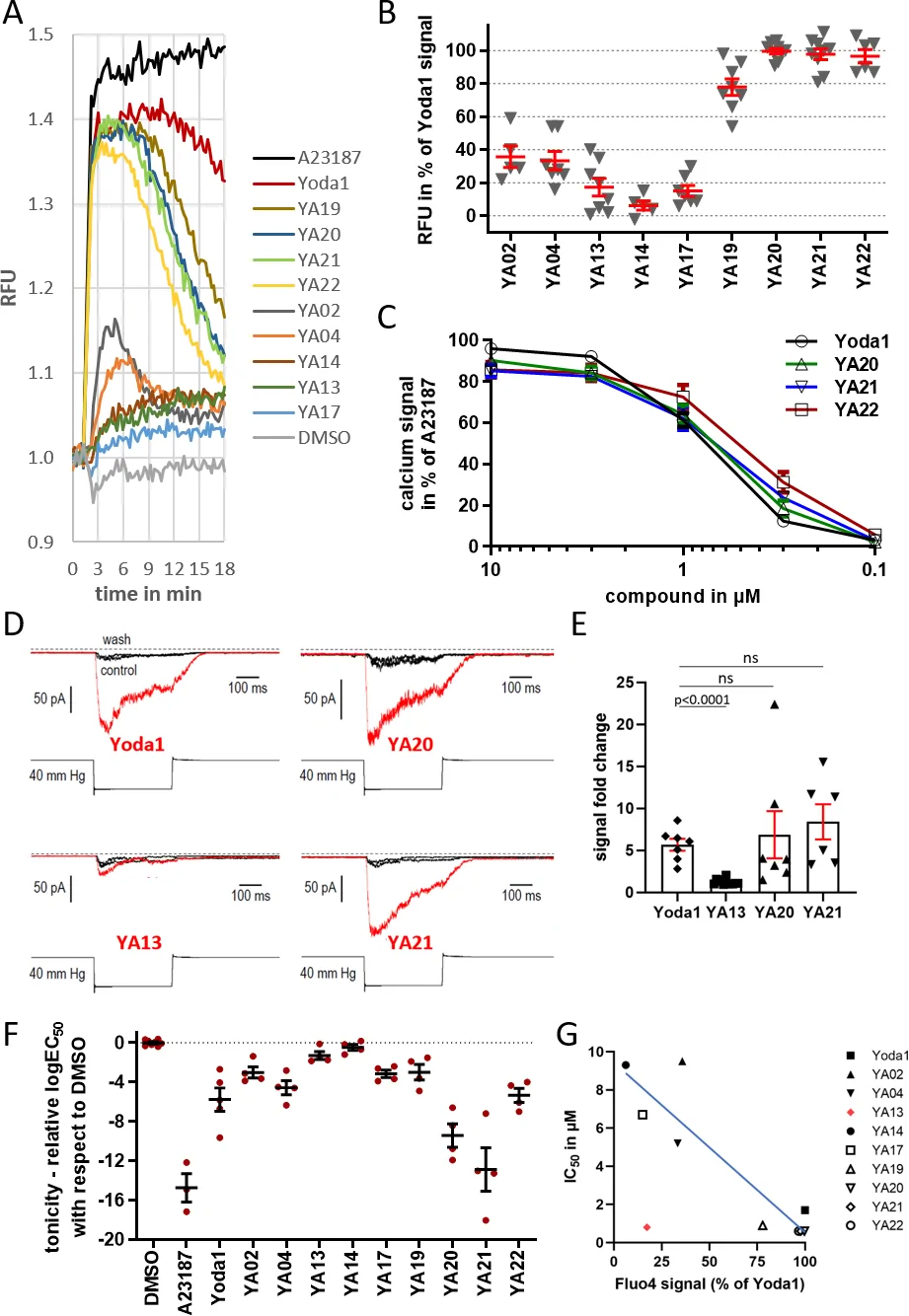
PIEZO1 sensitization by novel Yoda1 analogs. (**A**) Human RBC were pre-loaded with the calcium dye Fluo4-AM. Kinetic readings of fluorescence before and after the addition of compounds at 10 µM. The calcium ionophore A23187 (5 µM) was used as positive control. One representative experiment is shown. (**B**) Quantification of the Fluo4 signal with respect to readings with Yoda1 (set to 100%). Individual data of independent experiments (different blood donors) are shown, mean ± SEM. (**C**) Dose-effect curve of the calcium signal (in % of the A23187 response, *n* = 3, mean ± SEM). (**D**) Inside-out patch clamp recordings from mPiezo1 transfected HEK^Piezo1del^ cells. Currents were elicited with a fast pressure step of −40 mm Hg at a holding potential of −80 mV. Compounds (at 10 μM) were added for 1 min and then washed away for 1 min. (**E**) Fold increase in PIEZO1 currents shown in (**D**) (Non-parametric Mann and Whitney *U* test, ns = not significant). (**F**) Osmotic fragility assays of human RBC treated for 30 min with compounds at 10 µM. Negative logEC_50_ values relative to the DMSO control reflect dehydration. Data from different blood donors are shown, mean ± SEM. (**G**) Correlation of antibabesial activity with calcium-mobilization in human RBC. Simple linear regression was calculated after exclusion of the value for YA13 (in red). *R*^2^ = 0.84.

We then analyzed whether the new Yoda1 analogs can promote PIEZO1 opening in human RBC. We used compound-induced calcium influx as a proxy of PIEZO1 activation (Fig. 6A and B). Of the commercial compounds, YA02 and YA04 induced a weak transient signal (corresponding to about 30% of the Yoda1 effect), correlating with a lower protection against infection, as compared to Yoda1 (Fig. 2A). Signals for YA13, YA14, and YA17 were close to background levels (Fig. 6A and B) although YA13 exerted a potent antibabesial effect (Fig. 2A). In contrast, the newly synthesized analogs YA19–YA22, with improved specificity and lower toxicity, elicited a robust calcium signal, comparable to Yoda1 (Fig. 6B). The dose-effect curve for these analogs on the calcium signal was similar (Fig. 6C). YA20 and YA21 produced the most robust calcium signal (Fig. 6B) and were further analyzed regarding PIEZO1 activation (Fig. 6D and E). Electrophysiological characterization of YA13, YA20, and YA21 was performed using patch clamp experiments recorded in the excised inside-out configuration of PIEZO1-transfected HEK cells. While YA13 failed to activate PIEZO1 (Fig. 6D), in line with the absence of a Fluo4 calcium signal in RBC (Fig. 6B), YA20 and YA21 showed strong and reversible stimulation of PIEZO1 currents in response to pressure similar to Yoda1 (Fig. 6E). We also analyzed by osmotic fragility assays the effect of these new compounds on RBC dehydration as a consequence of PIEZO1 activity (5) (Fig. 6F). YA13 and YA14 that failed to elicit a calcium signal also did not induce RBC dehydration, further confirming that antibabesial activity of YA13 is unrelated to PIEZO1 activation. All other compounds induced dehydration to varying degrees, with YA20 and YA21 exerting the strongest effect (Fig. 6F).

## DISCUSSION

In summary, our results indicate that the newly synthesized Yoda1-like compounds YA19–YA22 are efficient PIEZO1 sensitizers with improved cytotoxicity/selectivity effects on *B. divergens* infection of human RBC *in vitro*. Notably, we observed a correlation between the antibabesial activity and the calcium signal in RBC indicating PIEZO1 activity, except for YA13 (in red) that probably exerts a deleterious effect on the parasite itself (Fig. 6G). Protection of human RBC by these compounds was superior against *B. divergens* infection, as compared to *P. falciparum*. Our previous findings indicated that *in vitro* protection against *P. falciparum* infection of human RBC is due to an early loss of biconcave discoid shape of human RBC, with secondarily a delayed RBC dehydration (9). A recent screening for drugs active against *B. divergens*, based on our previous work on malaria (9), also identified Yoda1 as a putative protective agent, among other pharmacological agents (13). In the present study, we further identified YA19–YA22 as novel and improved compounds against *Babesia* infection. These molecules show lower cell toxicity and higher specificity, as compared to *P. falciparum* infection. The metabolic stability of Yoda1 and YA20 in a standard mouse liver microsome assay revealed very short half-lives of 2 ± 1 min and 3.6 ± 0.2 min, respectively. Such short half-lives require further chemical optimizations or protective pharmacological formulations before their possible experimental evaluation in animal infection models.

Previous findings indicated that only minor chemical modifications are permitted on the Yoda1 molecule for the activity on PIEZO1 (8). These are replacing the thiadiazole ring by an oxadiazole (Dooku1 [12], Yaddle1 [14]), the pyrazine ring by a benzoic acid (KC289, Fig. 1), or a pyrrole (Dooku1 [to antagonize Yoda1-induced PIEZO1 channel activity]) (12). Here, we identified the substitution of the thioether linker by an ether (YA19 to YA22, Fig. 4) and the addition of a trifluoromethyl on the dichlorophenyl ring (YA22, Fig. 4). The structural constraints suggest that Yoda1 fits well into the allosteric binding site, leaving little room for changes in shape, with the optimal chemical modification being YA19–YA22, to improve toxicity index and specificity for *B. divergens*. For YA20 and YA21, acute application (1 min; Figure 6D and E) exerted a comparable effect to Yoda1 on PIEZO1 activation, while a chronic treatment showed improved antiparasitic properties (18 h; Fig. 2) together with a stronger RBC dehydration (30 min; Fig. 6F). These findings are compatible with an enhanced chemical stability for both YA20 and YA21. Our *in vitro* findings indicate that promoting PIEZO1 opening in human RBC with Yoda1-like molecules exerts a significant protection against *Babesia* infection.

## MATERIALS AND METHODS

### Biological material

*Babesia divergens* (strain Rouen 1987, clone 4) or *Plasmodium falciparum* (3D7) was cultured in human O^+^ or A^+^ erythrocytes in complete medium consisting of RPMI 1640 medium containing 25 mM HEPES and 2 mM L-glutamine (Gibco), 10% human AB + serum and 40 µg/mL gentamycin (Gibco). Human blood obtained as donations from anonymized individuals was provided by the local blood bank (Etablissement Français du Sang under the approval number 21PLER2016-0103). Parasitized RBC were maintained at 5% hematocrit at 37°C under a tri-gaseous mixture of 5% CO_2_, 5% O_2_, and 90% N_2_. Parasitemia was routinely monitored on thin blood smears fixed in methanol and stained with Diff-Quick (pH 7.2) (Dade Behring, France). For microscopic determination of parasitemia, at least 1,000 cells were counted.

### Determination of compound activities against *Babesia divergens* and *Plasmodium falciparum*

The effect of compounds on the growth of *B. divergens* and *P. falciparum* was measured in 96 well flat bottom microtiter plates according to a modified Desjardins test (15, 16) in complete medium. Infected RBC were grown at 1.5% final hematocrit and 1.5% parasitemia (for *B. divergens*) or 0.5% (for *P. falciparum*) and mixed with compounds or with DMSO solvent as control. Parasite growth was assessed by measuring the incorporation of [^3^H]hypoxanthine into nucleic acids as previously described (15). Suspensions of infected erythrocytes (200 µL) were mixed with a series of concentrations of the compounds and 30 μL of [^3^H]hypoxanthine in complete medium (0.5 μCi/well) were added directly. Incubation at 37°C under 5% CO_2_, 5% O_2_ was for 18 h for *B. divergens* corresponding to about three erythrocytic cycles (invasion, growth, and egress) in our culture conditions. Incubation was for 48 h for *P. falciparum*, corresponding to one complete erythrocytic cycle. The reactions were stopped and stored by freezing at −80°C. Cells were lysed by thawing, and the parasite macromolecules including nucleic acids were recovered by harvesting the lysate on glass-fiber filter plates (Unifilter 96 GF/C, PerkinElmer) using a FilterMate cell harvester (Packard Instruments). The radioactivity was counted on a MicroBeta 2 microplate scintillation counter (Perkin Elmer). Radioactivity background was obtained from incubation of uninfected RBC under the same conditions, and the value obtained was subtracted. Results were expressed as IC_50_, which is the drug concentration leading to 50% inhibition of parasite growth. A nonlinear regression model (sigmoidal dose–response/variable slope; GraphPad Prism 8) was used to calculate the IC_50_ values.

### *In vitro* toxicity against erythroblast cell line K562

K-562 cells were cultured in complete medium (RPMI 1640 with GlutaMAX, 10% human serum, 1× Penicillin/Streptomycin) in 96-well microplates (8,000 cells per well) and incubated for 24 h at 37°C under 5% CO_2_ in the presence of a series of concentrations of compounds. After incubation for 24 h at 37°C, 30 μL of complete medium containing 0.5 μCi [^3^H]thymidine was added to each well for an additional period of 6 h. The reactions were stopped by freezing at −80°C. Cell macromolecules including nucleic acids were recovered and quantified as described above for *Plasmodium*. Radioactivity background values were obtained from wells with incubation of culture medium in the absence of cells.

### Calcium measurements

Assays were performed in saline solution (145 mM NaCl, 4 mM KCl, 0.15 mM MgCl_2_, 2 mM CaCl_2_, 10 mM glucose, 10 mM HEPES). RBC were washed three times in saline and 5 × 10^7^ RBC were labeled with 5 µM Fluo4-AM (Invitrogen) and incubated for 30 min at 37°C. Cells were washed three times, resuspended in saline, and incubated for de-esterification for 30 min at room temperature. Suspensions of 1 × 10^6^ RBC in 100 µL were distributed in 96 well plates. Baseline fluorescence measurements were taken over 2 min at 20 s intervals in a SPARK multifunctional microplate reader (TECAN) with excitation at 485 nm and emission at 535 nm. Assays were then started by adding 100 µL of double concentrated drugs in saline and mixed using a multichannel pipette. Measurements were followed over 15 to 20 min. All values were divided by the mean of the sample before the addition of compounds. Fluorescent signals were presented as relative fluorescent units (RFU) with mock (DMSO) treated samples as negative control and A23187 calcium ionophore (10 µM) as positive control.

### Osmotic fragility assays

Osmotic fragility assays were performed as described (5) with minor modifications (17). Briefly, blood from different donors was used the day of reception whenever possible. The blood was washed twice in normal saline (NS, 149 mM NaCl, 2 mM HEPES, pH 7.4). Suspension of 1 mL blood at 2.5% hematocrit was incubated with compounds for 30 min at 37°C in in NS supplemented with 4 mM KCl and 2 mM CaCl_2_. Identical quantities of DMSO solvent served as controls. Suspension (10 μL) was then distributed in rows of U-bottom 96-well plates. Solutions of varying tonicity were generated by mixing NS (100%) with 2 mM HEPES, pH 7.4 (0%). Two hundred fifty microliters of these solutions was added to the diluted blood and incubated for 5 min at room temperature. Plates were then centrifuged at 1,000 *g* for 3 min, and 150 μL of the supernatant was transferred to flat bottom 96-well plates and centrifuged to remove air bubbles. Absorbance was measured using a SPARK multifunctional microplate reader (TECAN) at 415 nm. LogEC_50_ values were determined by fitting the data to 4-parameter sigmoidal dose–response curves using Prism (GraphPad).

### Electrophysiology analysis

HEK PIEZO1^del^ cells were plated on 35 mm non- or poly-L-lysine coated plastic dishes. HEK cells were recorded 48–72 h post-transfection. Data were acquired using an Axopatch 700A amplifier (Axon Instruments), Digidata 1322A digitizer (Molecular Devices), and pCLAMP10.7 software. Currents were sampled at 20 kHz and filtered at 1 kHz. All patch clamp recordings were performed in excised (inside-out) patch configurations at room temperature. Patch pipettes were made from 1.5 mm outer diameter glass (Vitrex Medical, #160214) using a Narishige PC-10 puller and had resistance of 1.2–1.4 MΩ when filled with a saline solution consisting of (in mM) 150 NaCl, 5 KCl, 2 CaCl_2_, 1 MgCl_2_, 10 HEPES (pH adjusted to 7.35 with NaOH) (External buffer). The bath solution consisted of (in mM) 155 KCl, 3 MgCl_2_, 5 EGTA, 10 HEPES (pH adjusted to 7.2 with KOH). The osmolality of all solutions was 310 mOsmol·kg^−1^. Membrane patches were subjected to stepwise, 350-ms long pressure pulses (Δ: 10 mm Hg) through the recording electrode using a fast pressure-clamp device (High Speed Pressure Clamp-1 system, ALA-scientific). Yoda1 and analogs were dissolved at 50 mM in DMSO and used at a final concentration of 10 μM. Drugs were applied by superfusion in the medium, and DMSO was added in the control solution.

### Statistical analysis

Data analysis and plots were generated using Excel (Microsoft) and Prism (GraphPad). Osmotic fragility curves were generated by fitting the data to a four-parameter sigmoidal dose–response curve, and LogEC_50_ values were calculated. Statistical significance was determined by non-parametric Mann–Whitney *U* test or by unpaired two-tailed *t*-test, depending on the size and normality of the population (GraphPad Prism). Means associated with the standard error of the mean (SEM) are illustrated on the figures, and details of statistical analyses are indicated in the legends. The number of independent experiments (*n*) is indicated throughout the manuscript.

### Metabolic stability (mouse liver microsomes)

Incubations in phosphate buffer containing 0.5 mg/mL of mouse liver microsomes were used to determine metabolic stability. The 10 mM compound stock solution in DMSO was diluted to a final incubation concentration of 1 μM. For cytochrome P450 reactions study, a NADPH regenerating system was used consisting of 1 mM NADP and 3 mM MgCl_2_. The resulting mixture was incubated at 37°C for various times up to 60 min. The incubation of each aliquot was stopped at 2, 10, 20, 40, and 60 min by adding 70 μL of ice-cold acetonitrile. Samples were vortexed and sonicated, and liquid chromatography-mass spectrometry was performed on the supernatant. The percentage of the remaining test compound relative to t0 was measured by monitoring the peak area on the chromatogram. An incubation was also performed in the absence of NADPH to reveal any chemical instability or non-NADPH-dependent enzymatic degradation and showed that Yoda1 and YA20 were stable. Testosterone was used as positive control. Half-life (t1/2) was estimated from the slope of the logarithmic curve of the compound remaining (%) against time, assuming first-order kinetics.

### Commercial identifier at Molport or Ambinter of commercial analogs of Yoda1

YA01 (MolPort-002-926-686), YA02 (MolPort-002-917-984), YA03 (MolPort-000-668-791), YA04 (MolPort-004-051-767), YA05 (MolPort-002-231-409), YA06 (MolPort-004-010-923), YA07 (MolPort-003-199-711), YA08 (MolPort-004-052-030), YA09 (MolPort-004-096-099), YA10 (MolPort-004-275-210), YA11 (MolPort-004-576-267), YA12 (MolPort-005-858-969), YA13 (MolPort-004-023-307), YA14 (MolPort-006-053-463), YA15 (MolPort-003-323-142), YA16 (MolPort-002-770-045), YA17 (MolPort-004-589-488), YA18 (Amb6646723).

### Chemical synthesis of Yoda1 analogs

The synthesis of Yoda1 analogs is shown in Fig. 5. In the first steps, 1,3,4-thiadiazole-2-amine derivatives were synthesized by reaction of acid chloride prepared from commercially available aromatic acid with amino thiourea, followed by cyclization under acid conditions. Aminethiadiazole derivatives were subsequently converted under Sandmeyer conditions with copper(II) bromide and tert-butyl nitrite to the corresponding bromothiadiazoles. Final compounds were obtained through Ullmann-type cross-coupling reactions with several substituted benzyl alcohols to generate the ether linker.

### General chemical procedures

2-Pyrazinecarboxylic acid (1 eq) was suspended in CH_2_Cl_2_. Oxalyl chloride (3 eq) and a catalytic amount of DMF were added. The solution was stirred for 4 h at room temperature (RT). The mixture was concentrated under reduced pressure to obtain an orange oil. The oil was subsequently dissolved in dry THF (10 mL) and added portion-wise to a suspension of hydrazinecarbothioamide (2.2 eq) in THF (30 mL) at 0°C. The resulting suspension was stirred over night at RT under nitrogen atmosphere. The suspension was quenched with water and alkalized using 1N NaOH solution and extracted with ethyl acetate (three times). The combined ethyl acetate layers were dried over MgSO_4_, filtered, and concentrated to dryness. The crude residue was purified by flash chromatography on a silica gel column using a mixture of AcOEt/MeOH (95/5) to provide 2-(pyrazine-2-carbonyl)hydrazine-1-carbothioamide in 72% yield.

2-(pyrazine-2-carbonyl)hydrazine-1-carbothioamide was dissolved in sulfuric acid. The mixture was stirred for 3 h at 100°C. The reaction mixture was slowly poured into water and basified using NaOH solution 50% while cooling on an ice bath and extracted with ethyl acetate (three times). The combined ethyl acetate layers were dried over MgSO_4_, filtered, and concentrated to dryness. The crude residue was purified by flash chromatography on a silica gel column using a mixture of AcOEt/MeOH (95/5) to provide 5-(pyrazin-2-yl)−1,3,4-thiadiazol-2-amine in 82% yield.

5-(pyrazin-2-yl)−1,3,4-thiadiazol-2-amine (1 eq) was added portion-wise over 5 min to a stirred solution of CuBr_2_ (2.2 eq) and t-BuNO_2_ (2.2 eq) in MeCN under nitrogen at 0°C, and the reaction was stirred over night at 65°C. Then, the reaction mixture was slowly poured into water and basified using NaOH solution 50% while cooling on an ice bath and extracted with ethyl acetate (three times). The combined ethyl acetate layers were dried over MgSO_4_, filtered, and concentrated to dryness. The crude residue was purified by flash chromatography on a silica gel column using a mixture of AcOEt/cyclohexane (4/6) to provide 2-bromo-5-(pyrazin-2-yl)−1,3,4-thiadiazole in 82% yield.

To a solution of 2-bromo-5-(pyrazin-2-yl)−1,3,4-thiadiazole (1 eq) in dry THF under argon at 0°C NaH (2,2 eq) was added. After 1 h stirring at room temperature, KI (1 eq), CuO (1 eq), and the alcohol (1 eq) were added, and the reaction was stirred for 20 min at 50°C under microwave. Then, the reaction mixture was washed with brine and concentrated to dryness. The crude residue was purified by flash chromatography on a silica gel column using a mixture of AcOEt/cyclohexane to provide the desired product.

### ^1^H nuclear magnetic resonance spectra for YA19, YA20, YA21, and YA22 (Fig. 5)

#### YA19: 2-((2,6-dichlorobenzyl)oxy)-5-(pyrazin-2-yl)-1,3,4-thiadiazole

^1^H NMR (200 MHz, CDCl_3_) δ 9.49 (d, *J* = 1.5 Hz, 1H), 8.71–8.49 (m, 2H), 7.36 (m, 3H), 5.92 (s, 2H).

^13^C NMR (50 MHz, CDCl_3_) δ 176.49, 162.54, 145.39, 145.26, 143.94, 141.88, 137.34, 131.30, 130.30, 128.55, 69.36.

MS *m*/*z* (ESI) 338.80 (M + H)^+^.

#### YA20: 2-((2-chloro-6-(trifluoromethyl)benzyl)oxy)-5-(pyrazin-2-yl)-1,3,4-thiadiazole

^1^H NMR (200 MHz, CDCl_3_) δ 9.48 (d, *J* = 1.5 Hz, 1H), 8.69–8.48 (m, 2H), 7.77-7.60 (m, 2H), 7.60–7.40 (m, 1H), 5.88 (s, 2H).

^13^C NMR (50 MHz, CDCl_3_) δ 176.26 (s), 162.70 (s), 145.35 (d, *J* = 12.9 Hz), 143.71 (d, *J* = 25.5 Hz), 141.90 (d, *J* = 21.5 Hz), 138.60 (s), 133.76 (s), 133.37–131.32 (m), 130.84 (s), 130.27 (s), 126.05 (s), 125.29–123.60 (m), 120.59 (s), 67.69 (s).

MS *m*/*z* (ESI) 372.93 (M + H)^+^.

#### YA21: 2-((2,6-dichloro-3-(trifluoromethyl)benzyl)oxy)-5-(pyrazin-2-yl)-1,3,4-thiadiazole

^1^H NMR (200 MHz, CDCl_3_) δ 9.49 (d, *J* = 1.5 Hz, 1H), 8.67–8.53 (m, 2H), 7.72 (d, *J* = 8.6 Hz, 1H), 7.52 (d, *J* = 8.6 Hz, 1H), 5.98 (s, 2H).

^13^C NMR (50 MHz, CDCl_3_) δ 176.24 (s), 162.91 (s), 145.35 (d, *J* = 20.1 Hz), 144.01 (s), 143.50 (s), 141.92 (d, *J* = 18.7 Hz), 141.13 (s), 135.87 (s), 132.76 (s), 129.25 (s), 128.35 (t, *J* = 15.9 Hz), 125.05 (s), 119.61 (s), 68.77 (s).

MS *m*/*z* (ESI) 406.93 (M + H)^+^.

#### YA22: 2-((2,6-bis(trifluoromethyl)benzyl)oxy)-5-(pyrazin-2-yl)-1,3,4-thiadiazole

^1^H NMR (200 MHz, CDCl_3_) δ 9.50 (d, *J* = 1.5 Hz, 1H), 8.72–8.52 (m, 2H), 8.02 (d, *J* = 8.0 Hz, 2H), 7.73 (t, *J* = 8.0 Hz, 1H), 5.89 (s, 2H).

^13^C NMR (50 MHz, CDCl_3_) δ 175.82 (s), 162.81 (s), 145.36 (d, *J* = 13.5 Hz), 143.96 (s), 141.94 (s), 133.73 (s), 133.11 (s), 132.49 (s), 131.08 (s), 130.21 (d, *J* = 6.4 Hz), 126.10 (s), 120.63 (s), 66.13 (s).

MS *m*/*z* (ESI) 406.93 (M + H)^+^.

## Supplementary Material

Reviewer comments

## Data Availability

All data generated or analyzed during this study are included in this published article.

## SUPPLEMENTAL MATERIAL

OPEN PEER REVIEW reviewer-comments.pdf An accounting of the reviewer comments and feedback.

ASM does not own the copyrights to Supplemental Material that may be linked to, or accessed through, an article. The authors have granted ASM a non-exclusive, world-wide license to publish the Supplemental Material files. Please contact the corresponding author directly for reuse.
