# Supplementary material for: Protection against Babesia infection by red blood cell PIEZO1 activation
Source: Microbiol Spectr. 2026 Jul 29;14(9):e04114-25. doi: 10.1128/spectrum.04114-25 (PMC13532334; doi:10.1128/spectrum.04114-25)
Supplement: Supplementary file 1 [file reviewer-comments.pdf]

AMERICAN SOCIETY FOR MICROBIOLOGY | Microbiology Spectrum

Protection against *Babesia* infection by red blood cell PIEZO1 activation

Kai WENGELNIK, Rakhee LOHIA, Anais PAVEYRANNE, Jordy LE GUET, Gregor JOSE, Stephane AZOULAY, Eric HONORE, and Dominique DOUGUET

Corresponding Author(s): Dominique DOUGUET, Institut de Pharmacologie Moleculaire et Cellulaire

Review Timeline:

Submission Date:	December 18, 2025
Editorial Decision:	March 30, 2026
Revision Received:	March 31, 2026
Editorial Decision:	May 15, 2026
Revision Received:	May 29, 2026
Accepted:	June 14, 2026

Editor: Anna Moniuszko-Malinowska

Reviewer(s): Disclosure of reviewer identity is with reference to reviewer comments included in decision letter(s). The following individuals involved in review of your submission have agreed to reveal their identity: Jinming Wang (Reviewer #2)

Transaction Report:

(Note: With the exception of the correction of typographical or spelling errors that could be a source of ambiguity, letters and reports are not edited. The original formatting of letters and referee reports may not be reflected in this compilation.)

DOI: https://doi.org/10.1128/spectrum.04114-25

Re: Spectrum04114-25 (**Protection against *Babesia* infection by red blood cell PIEZO1 activation**)

Dear Dr. Dominique DOUGUET:

Thank you for the privilege of reviewing your work. Below you will find my comments, instructions from the Spectrum editorial office, and the reviewer comments.

Please return the manuscript within 60 days; if you cannot complete the modification within this time period, please contact me. If you do not wish to modify the manuscript and prefer to submit it to another journal, notify me immediately so that the manuscript may be formally withdrawn from consideration by Spectrum.

Revision Guidelines
To submit your modified manuscript, log into the submission site at https://spectrum.msubmit.net/cgi-bin/main.plex. Go to Author Tasks and click the appropriate manuscript title to begin. The information you entered when you first submitted the paper will be displayed; update this as necessary. Note the following requirements:

• Upload point-by-point responses to the issues raised by the reviewers in a file named "Response to Reviewers," NOT in your cover letter.
• Upload a compare copy of the manuscript (without figures) as a "Marked-Up Manuscript" file.
• Upload a clean .DOC/.DOCX version of the revised manuscript and remove the previous version.
• Each figure must be uploaded as a separate, editable, high-resolution file (TIFF or EPS preferred), and any multipanel figures must be assembled into one file.
• Any supplemental material intended for posting by ASM should be uploaded with their legends separate from the main manuscript. You can combine all supplemental material into one file (preferred) or split it into a maximum of 10 files with all associated legends included.

For complete guidelines on revision requirements, see our Submission and Review Process webpage. Submission of a paper that does not conform to guidelines may delay acceptance of your manuscript.

Data availability: ASM policy requires that data be available to the public upon online posting of the article, so please verify all links to sequence records, if present, and make sure that each number retrieves the full record of the data. If a new accession number is not linked or a link is broken, provide Spectrum production staff with the correct URL for the record. If the accession numbers for new data are not publicly accessible before the expected online posting of the article, publication may be delayed; please contact production staff (Spectrum@asmusa.org) immediately with the expected release date.

Publication Fees: For information on publication fees and which article types are subject to charges, visit our website. If your manuscript is accepted for publication and any fees apply, you will be contacted separately about payment during the production process; please follow the instructions in that e-mail. Arrangements for payment must be made before your article is published.

ASM Membership: Corresponding authors may join or renew ASM membership to obtain discounts on publication fees. Need to upgrade your membership level? Please contact Customer Service at Service@asmusa.org.

The ASM Journals program strives for constant improvement in our submission and publication process. Please tell us how we can improve your experience by taking this quick Author Survey.

Thank you for submitting your paper to Spectrum.

Sincerely,
Anna Moniuszko-Malinowska
Editor
Microbiology Spectrum

Reviewer #1 (Comments for the Author):

In this manuscript, the authors have tested vitro protection effect of human erythrocytes against Babesia divergens by the PIEZO1 chemical sensitizer Yoda1 and derivatives, and found that a newly synthesized molecule named YA20 exhibits improved submicromolar antiparasitic properties against B. divergens and with reduced cytotoxicity. The study has demonstrated the proof-of-concept of antibabesial potential of using PIEZO1 chemical sensitizers. The reviewer has the following minor comments, which might require the authors for clarification or discussion.

1) Please state the rational for using the mammalian lymphoblast cell line K562 as the cell type for testing the cytotoxicity of Piezo1 activators shown in Table 1.
2) in Figure 2B-E, please justify why YA19 and YA22 were not functionally tested.
3) Based on the functional characterization, YA20 and YA21 show similar effect in affecting Piezo1-mediated Ca2+ signal and mechanically evoked current. However, in the osmotic fragility assays of human RBCs, YA20 and YA21 show stronger effect than Yoda1 (as well as Ya19 and YA22). Please provide an explanation.

March 31th, 2026

Re: Spectrum04114-25 (**Protection against *Babesia* infection by red blood cell PIEZO1 activation**)

Dear Dr. Dominique DOUGUET:

Thank you for the privilege of reviewing your work. Below you will find my comments, instructions from the Spectrum editorial office, and the reviewer comments.

Please return the manuscript within 60 days; if you cannot complete the modification within this time period, please contact me. If you do not wish to modify the manuscript and prefer to submit it to another journal, notify me immediately so that the manuscript may be formally withdrawn from consideration by Spectrum.

Thank you for submitting your paper to Spectrum.

Sincerely,
Anna Moniuszko-Malinowska
Editor
Microbiology Spectrum

Reply:

Thank you very much for providing the opportunity to respond to the reviewers' comments.

Reviewer comments:

Reviewer #1 (Comments for the Author):

In this manuscript, the authors have tested vitro protection effect of human erythrocytes against Babesia divergens by the PIEZO1 chemical sensitizer Yoda1 and derivatives, and found that a newly synthesized molecule named YA20 exhibits improved submicromolar antiparasitic properties against B. divergens and with reduced cytotoxicity. The study has demonstrated the proof-of-concept of antibabesial potential of using PIEZO1 chemical sensitizers. The reviewer has the following minor comments, which might require the authors for clarification or discussion.

We would like to thank Reviewer 1 for its very helpful suggestions.

1) Please state the rational for using the mammalian lymphoblast cell line K562 as the cell type for testing the cytotoxicity of Piezo1 activators shown in Table 1.

Reply:

We updated the text to indicate that K-562 lymphoblast erythroid cells were selected for cytotoxicity assays because they can grow as cell suspension, similarly to *P. falciparum* culture in human RBC.

2) in Figure 2B-E, please justify why YA19 and YA22 were not functionally tested.

Reply:

We now added a sentence to clarify our selection of compounds YA20 and YA21. Indeed, they produced the most robust calcium signal (Figure 2B) and thus, were further analyzed regarding PIEZO1 activation (Figure 2D-E).

3) Based on the functional characterization, YA20 and YA21 show similar effect in affecting Piezo1-mediated Ca2+ signal and mechanically evoked current. However, in the osmotic fragility assays of human RBCs, YA20 and YA21 show stronger effect than Yoda1 (as well as Ya19 and YA22). Please provide an explanation.

Reply:

We now added a comment in the Discussion that indicates that YA20 and YA21, acute application (1 minute; Fig. 2D-E) exerted a comparable effect to Yoda1 on PIEZO1 activation, while a chronic treatment showed improved antiparasitic properties (18 hours; Fig. 1) together with a stronger RBC dehydration (30 minutes; Fig. 2F). These findings are compatible with an enhanced chemical stability for both YA20 and YA21.

Re: Spectrum04114-25R1 (**Protection against *Babesia* infection by red blood cell PIEZO1 activation**)

Dear Dr. Dominique DOUGUET:

Thank you for the privilege of reviewing your work. Below you will find my comments, instructions from the Spectrum editorial office, and the reviewer comments.

Please return the manuscript within 60 days; if you cannot complete the modification within this time period, please contact me. If you do not wish to modify the manuscript and prefer to submit it to another journal, notify me immediately so that the manuscript may be formally withdrawn from consideration by Spectrum.

Revision Guidelines
To submit your modified manuscript, log into the submission site at https://spectrum.msubmit.net/cgi-bin/main.plex. Go to Author Tasks and click the appropriate manuscript title to begin. The information you entered when you first submitted the paper will be displayed; update this as necessary. Note the following requirements:

• Upload point-by-point responses to the issues raised by the reviewers in a file named "Response to Reviewers," NOT in your cover letter.
• Upload a compare copy of the manuscript (without figures) as a "Marked-Up Manuscript" file.
• Upload a clean .DOC/.DOCX version of the revised manuscript and remove the previous version.
• Each figure must be uploaded as a separate, editable, high-resolution file (TIFF or EPS preferred), and any multipanel figures must be assembled into one file.
• Any supplemental material intended for posting by ASM should be uploaded with their legends separate from the main manuscript. You can combine all supplemental material into one file (preferred) or split it into a maximum of 10 files with all associated legends included.

For complete guidelines on revision requirements, see our Submission and Review Process webpage. Submission of a paper that does not conform to guidelines may delay acceptance of your manuscript.

Data availability: ASM policy requires that data be available to the public upon online posting of the article, so please verify all links to sequence records, if present, and make sure that each number retrieves the full record of the data. If a new accession number is not linked or a link is broken, provide Spectrum production staff with the correct URL for the record. If the accession numbers for new data are not publicly accessible before the expected online posting of the article, publication may be delayed; please contact production staff (Spectrum@asmusa.org) immediately with the expected release date.

Publication Fees: For information on publication fees and which article types are subject to charges, visit our website. If your manuscript is accepted for publication and any fees apply, you will be contacted separately about payment during the production process; please follow the instructions in that e-mail. Arrangements for payment must be made before your article is published.

ASM Membership: Corresponding authors may join or renew ASM membership to obtain discounts on publication fees. Need to upgrade your membership level? Please contact Customer Service at Service@asmusa.org.

The ASM Journals program strives for constant improvement in our submission and publication process. Please tell us how we can improve your experience by taking this quick Author Survey.

Thank you for submitting your paper to Spectrum.

Sincerely,
Anna Moniuszko-Malinowska
Editor
Microbiology Spectrum

Reviewer #1 (Comments for the Author):

The authors have addressed the comments. The reviewer supports the publication of the manuscript.

Reviewer #2 (Comments for the Author):

Reviewer Comments
The manuscript "Protection against Babesia infection by red blood cell PIEZO1
activation" presents potentially important data for controlling babesiosis. However, several concerns need to be addressed before it can be accepted for publication. In vivo efficacy of Yoda1.
The authors have shown that several Yoda1 analogs possess activity against Babesia divergens in vitro. To strengthen the study, the authors should further confirm whether the parent compound Yoda1 (or its analogs) is effective in vivo, for example, using a BALB/c mouse model infected with Babesia microti.

Reviewer Comments

The manuscript “Protection against *Babesia* infection by red blood cell PIEZO1 activation” presents potentially important data for controlling babesiosis. However, several concerns need to be addressed before it can be accepted for publication.

In vivo efficacy of Yoda1.

The authors have shown that several Yoda1 analogs possess activity against *Babesia divergens* in vitro. To strengthen the study, the authors should further confirm whether the parent compound Yoda1 (or its analogs) is effective in vivo, for example, using a BALB/c mouse model infected with *Babesia microti*.

Reviewer Comments

The manuscript “Protection against Babesia infection by red blood cell PIEZO1 activation” presents potentially important data for controlling babesiosis. However, several concerns need to be addressed before it can be accepted for publication.

In vivo efficacy of Yoda1.

The authors have shown that several Yoda1 analogs possess activity against Babesia divergens in vitro. To strengthen the study, the authors should further confirm whether the parent compound Yoda1 (or its analogs) is effective in vivo, for example, using a BALB/c mouse model infected with Babesia microti.

Reply to reviewer 2:

We entirely agree with the reviewer that the *in vivo* evaluation of our compounds would certainly be valuable. However, when we assessed the metabolic stability of Yoda1 using mouse liver microsomes the compound was found to have a very short half-life of2 min. Our compound YA20 shows a half-life that is almost doubled (3.6 min). However, this remains obviously too short to technically and ethically justify *in vivo* animal experiments.

We now inserted the following lines in the discussion (line 114 ff):

“The metabolic stability of Yoda1 and YA20 in a standard mouse liver microsome assay revealed very short half-lives of 2 ± 1 min and 3.6 ± 0.2 min, respectively. Such short half-lives require further chemical optimizations or protective pharmacological formulations before their possible experimental evaluation in animal infection models.”

Re: Spectrum04114-25R2 (**Protection against *Babesia* infection by red blood cell PIEZO1 activation**)

Dear Dr. Dominique DOUGUET:

Your manuscript has been accepted, and I am forwarding it to the ASM production staff for publication. Your paper will first be checked to make sure all elements meet the technical requirements. ASM staff will contact you if anything needs to be revised before copyediting and production can begin. Otherwise, you will be notified when your proofs are ready to be viewed.

Data Availability: ASM policy requires that data be available to the public upon online posting of the article, so please verify all links to sequence records, if present, and make sure that each number retrieves the full record of the data. If a new accession number is not linked or a link is broken, provide production staff with the correct URL for the record. If the accession numbers for new data are not publicly accessible before the expected online posting of the article, publication may be delayed; please contact ASM production staff immediately with the expected release date.

Publication Fees: For information on publication fees and which article types have charges, please visit our website. We have partnered with Copyright Clearance Center (CCC) to collect author charges. If fees apply to your paper, you will receive a message from no-reply@copyright.com with further instructions. For questions related to paying charges through RightsLink, please contact CCC at ASM_Support@copyright.com or toll free at +1-877-622-5543. CCC makes every attempt to respond to all emails within 24 hours.

ASM Membership: Corresponding authors may join or renew ASM membership to obtain discounts on publication fees. Need to upgrade your membership level? Please contact Customer Service at Service@asmusa.org.

Embargo Policy: A press release may be issued as soon as the manuscript is posted on the Spectrum Latest Articles webpage. The corresponding author will receive an email with the subject line "ASM Journals Author Services Notification" when the article is available online.

The ASM Journals program strives for constant improvement in our submission and publication process. Please tell us how we can improve your experience by taking this quick Author Survey.

Thank you for submitting your paper to Spectrum.

Sincerely,
Anna Moniuszko-Malinowska
Editor
Microbiology Spectrum
